# Electrophysiological evidence for pre-attention information processing improvement in patients with central hemiplegic after peripheral nerve rewiring: a pilot study

**DOI:** 10.1038/s41598-017-07263-z

**Published:** 2017-07-31

**Authors:** Tie Li, Xu-Yun Hua, Mou-Xiong Zheng, Yu-Lan Zhu, Yan-Qun Qiu, Yun-Dong Shen, Jian-Guang Xu, Yu-Dong Gu, Wen-Dong Xu

**Affiliations:** 1 Department of Hand Surgery, Huashan Hospital, Shanghai Medical College, Fudan University, Shanghai, China; 2 Department of Rehabilitation, Huashan Hospital, Shanghai Medical College, Fudan University, Shanghai, China; 3 Department of Hand and Upper Extremity Surgery, Jing’an District Central Hospital, Shanghai, China

## Abstract

Central neurologic injury (CNI) causes dysfunctions not only in limbs but also in cognitive ability. We applied a novel peripheral nerve rewiring (PNR) surgical procedure to restore limb function. Here, we conducted a prospective study to develop estimates for the extent of preattentive processes to cognitive function changes in CNI patients after PNR. Auditory mismatch negativity (MMN) was measured in CNI patients who received the PNR surgery plus conventional rehabilitation treatment. During the 2-year follow-up, the MMN was enhanced with increased amplitude in the PNR plus rehabilitation group compared to the rehabilitation-only group as the experiment progressed, and progressive improvement in behavioural examination tests was also observed. Furthermore, we found a significant correlation between the changes in Fugl-Meyer assessment scale scores and in MMN amplitudes. These results suggested that PNR could affect the efficiency of pre-attention information processing synchronously with the recovery of motor function in the paralyzed arm of the in chronic CNI patients. Such electroencephalographic measures might provide a biological approach with which to distinguish patient subgroups after surgery, and the change in MMN may serve as an objective auxiliary index, indicating the degree of motor recovery and brain cognitive function.

## Introduction

Central neurologic injury (CNI) is a major contributor to physical disability and can affect both adults and children. In addition to motor-sensory functional impairments, patients frequently suffer from severe neurocognitive dysfunction, including damaged motor execution abilities and decreased automatic information processing of the injured cortex. The disorder mainly affects patients’ muscle tone, movement, and daily living skills with varied levels of limb dysfunction and cognitive impairment^1^. For central hemiplegic patients, dysfunctions of sensory and motor information processing accompany limb dysfunction^2, 3^. These deficits of cognitive ability are potentially related to the difficulty of motion execution^4^, which may be an important obstacle that influences rehabilitation after CNI.

For hemiplegic patients, we applied a new surgical procedure of peripheral nerve rewiring to restore the motor function of the affected upper extremity. During the procedure, the nerve fibres that originally innervated the intact upper extremity were severed and transferred to the C7 nerve of the paralyzed arm^5^. This crossed peripheral nerve (contralateral C7, CC7) then connected the paralyzed upper extremity with the contra-lesional (ipsilateral to the paralyzed arm) hemisphere. The control of the contra-lesional hemisphere over the paralyzed upper extremity was enhanced, promoting brain plasticity. In the latest follow-up visit 2 years after surgery in the preliminary cases, the results were quite encouraging^6^. However, the relationship between motor recovery and cognitive changes remains a matter of debate, and more specifically, it is also unclear whether the brain plasticity process after peripheral nerve rewiring affects cognitive function.

Event-related potential (ERP) testing is a useful tool for the on-line examination of both normal and impaired information processing, and such research may contribute to the development of theoretical models of interaction between motor and cognitive function. The adequate processing of task-irrelevant deviant events is an important cognitive function for human survival^7–9^ and can be reflected by the mismatch negativity (MMN) component of ERPs. The precise neural function that is reflected by the MMN is controversial. Early studies suggested that the MMN reflects a preattentive, memory-based auditory discrimination process^7, 10^. However, it has recently been suggested that the MMN may reflect a much simpler neural adaption process.

Particularly relevant to the present study are a growing number of CNI (stroke, cerebral trauma, and cerebral palsy) studies that have investigated preattentive automatic processing by recording the auditory MMN. For example, Deouell *et al*.^4^ used the MMN to investigate the discrimination of different sound features in patients with right hemisphere damage that caused a neglect to the left hemi-field and found that an early deficit in detecting changes in the environment hampers the involuntary triggering of attention in these patients. Ilvonen *et al*.^11, 12^ investigated cognitive function in left-hemisphere-stroke patients and found a significant correlation between the change in the duration of MMN amplitude and speech-comprehension performance after stroke, suggesting that the MMN can be used as an index for the recovery of auditory discrimination.

To improve the cognitive function of CNI patients, psycho-physiological and cognitive behavioural training methods have been widely used. For example, there is evidence that frequent exposure to music is able not only to increase evoked potentials^13^ and gating^14^ in the auditory cortex but also to enhance learning, memory and neuronal plasticity in the animal brain^15^. Moreover, multimodal sensory stimulation has also been shown to reduce lesion volume and to improve cognitive and motor recovery after brain injury in rats^16^. Similarly, it has been reported that merely listening to music and speech after neural damage can induce long-term plastic changes in early sensory processing, which may facilitate the recovery of higher cognitive functions^17^. However, it is unclear whether the cortical plastic changes that occur after peripheral nerve rewiring are able to affect cognitive function. As described above, in humans, MMN can be used to measure crucial cortical plastic changes caused by training or remediation programmes^10, 18^. In the present study, during the recovery process of central hemiplegic patients after PNR, brain plasticity is an important component of functional restoration as well as peripheral nerve regeneration^19, 20^.We hypothesize that the coordination of the whole paralyzed upper extremity is improved in well-recovered patients. This improvement of motor control must inevitably involve the recovery of cognitive function. To test this hypothesis, we analysed the auditory MMN in patients after PNR with a follow-up period of 2 years.

## Results

**Figure 1:**
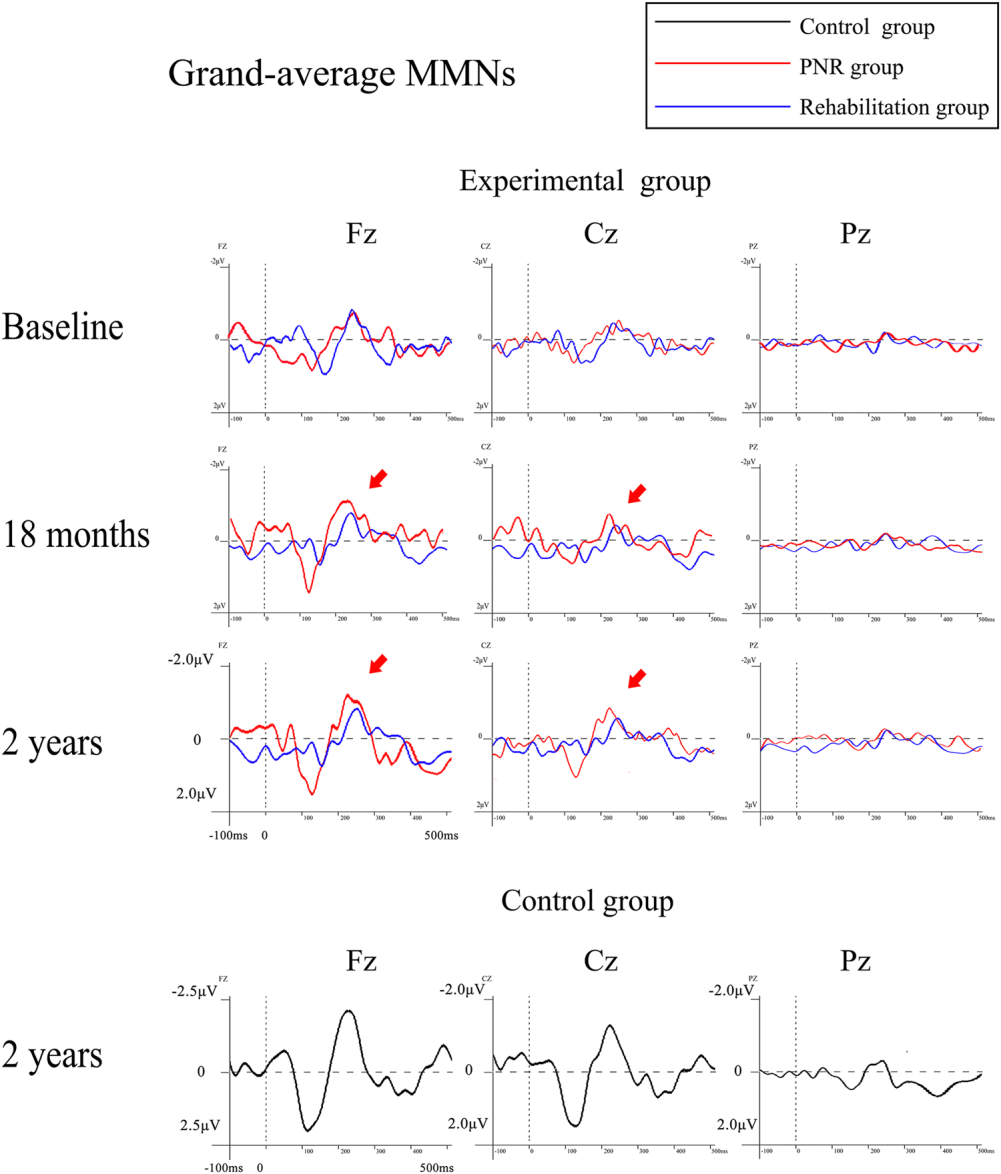
The grand mean averaged mismatch negativity (MMN) waveforms of the electrodes of Fz, Cz and Pz electrodes in the peripheral nerve rewiring (PNR) plus rehabilitation, rehabilitation-only and control groups.

Attenuation in the MMN amplitude was observed in both the PNR plus rehabilitation and the rehabilitation-only central hemiplegic patients. Figure 1 shows the averaged MMN waveforms of the Fz, Cz and Pz electrodes. The mean amplitudes of the MMNs are presented in Table 1.

**Table 1:** Mean Amplitudes (µV) (M ± SD) of mismatch negativity (MMN) at Fz, Cz, and Pz in central neurological injury (CNI) Patients and Control Subjects.

		PNR plus rehabilitation group	Rehabilitation-only group	Control group
Fz	Baseline	−0.77(±0.17)	−0.75(±0.23)	−1.99(±0.40)
	6 months	−0.81(±0.14)	−0.78(±0.25)	−1.99(±0.54)
	1 year	−0.84(±0.12)	−0.78(±0.25)	−1.96(±0.46)
	18 months	−1.12(±0.28)^ab^	−0.80(±0.14)	−2.06(±0.45)
	2 years	−1.18(±0.32)^ab^	−0.85(±0.15)	−2.02(±0.47)
Cz	Baseline	−0.53(±0.16)	−0.55(±0.12)	−1.33(±0.37)
	6 months	−0.57(±0.23)	−0.57(±0.18)	−1.26(±0.20)
	1 year	−0.66(±0.15)	−0.51(±0.23)	−1.24(±0.25)
	18 months	−0.75(±0.11)^ab^	−0.59(±0.21)	−1.31(±0.18)
	2 years	−0.81(±0.19)^ab^	−0.53(±0.24)	−1.42(±0.25)
Pz	Baseline	−0.24(±0.04)	−0.24(±0.04)	−0.34(±0.12)
	6 months	−0.26(±0.03)	−0.24(±0.05)	−0.32(±0.09)
	1 year	−0.24(±0.04)	−0.25(±0.03)	−0.32(±0.12)
	18 months	−0.26(±0.11)	−0.24(±0.05)	−0.33(±0.10)
	2 years	−0.25(±0.08)	−0.24(±0.05)	−0.33(±0.10)

The superscript (^a^) (P < 0.05) denotes significant differences between the surgery and rehabilitation-only groups. The superscript (^b^) (P < 0.05) denotes significant differences between the pretreatment and posttreatment values.

### MMN Significance

After visual inspection of the waveforms, we performed three-way repeated measures analysis on the individual Group (PNR, rehabilitation and control) *_Time (pre, post 6 months, post 1 year, post 18 months, post 2 years) *Electrodes (Fz, Cz and Pz), and the analysis yielded the following conclusions regarding the main effects of the three factors.

In the ANOVA analysis of MMN amplitudes, there was a significant main effect of Group [F(2, 54) = 871.2, p < 0.001,η² = 0.970], revealing that the MMN in normal healthy controls (−1.2 μV) was larger than that in the PNR plus rehabilitation group (−0.62 μV; p < 0.01), which was larger than that in the rehabilitation-only group (−0.50 μV; p < 0.05). These results show that the condition of the PNR plus rehabilitation group was significantly better than that of patients treated with rehabilitation only, but both groups were still different from the normal control group.

In the ANOVA analysis of MMN amplitudes, there was a significant main effect of time [F(2,53) = 1691.4, p =  < 0.001,η² = 0.985]. Further analyses revealed that the amplitude of MMN was significantly higher at 18 months (−0.83 μV; p < 0.01) and 2 years (−0.85 μV; p < 0.01) after PNR than before the procedure (−0.75 μV; p < 0.01).

In the ANOVA analysis of MMN amplitudes, there was a significant main effect of electrode site [F(4,51) = 10.7, p = 0.000,η² = 0.456], showing that the MMN in Fz (−1.2 μV) was larger than that in Cz (−0.8 μV; p < 0.01), which was larger than that in Pz (−0.3 μV; p < 0.01).

Interestingly, we found a significant two-way interaction of Group * Time, [F(8, 216) = 4.2, p < 0.001,η² = 0.134]. Further analysis of this interaction showed that there was a significant time effect for the PNR plus rehabilitation group (p < 0.05), whereas the time effect was not significant for either normal controls (p = 0.17) or rehabilitation-only group (p = 0.86). Although the group effect was evident for each time point (p < 0.005 in all cases), the MMN found in the PNR plus rehabilitation group was larger than that in the rehabilitation-only group only in the 4^th^ (−0.71 μV and −0.54 μV for PNR plus rehabilitation and rehabilitation groups, respectively; p < 0.001) and 5^th^ (−0.75 μV and −0.54 μV for PNR plus rehabilitation and rehabilitation groups, respectively; p < 0.001) time points. No other effects reached significant level (p > 0.1 in all cases).

For the MMN components, the MMN peak was not easily discernible at all sites and in each condition in many participants, the analysis was based on the mean amplitude calculated between control and oddball MMNs.

### Behavioural Assessments

**Figure 2:**
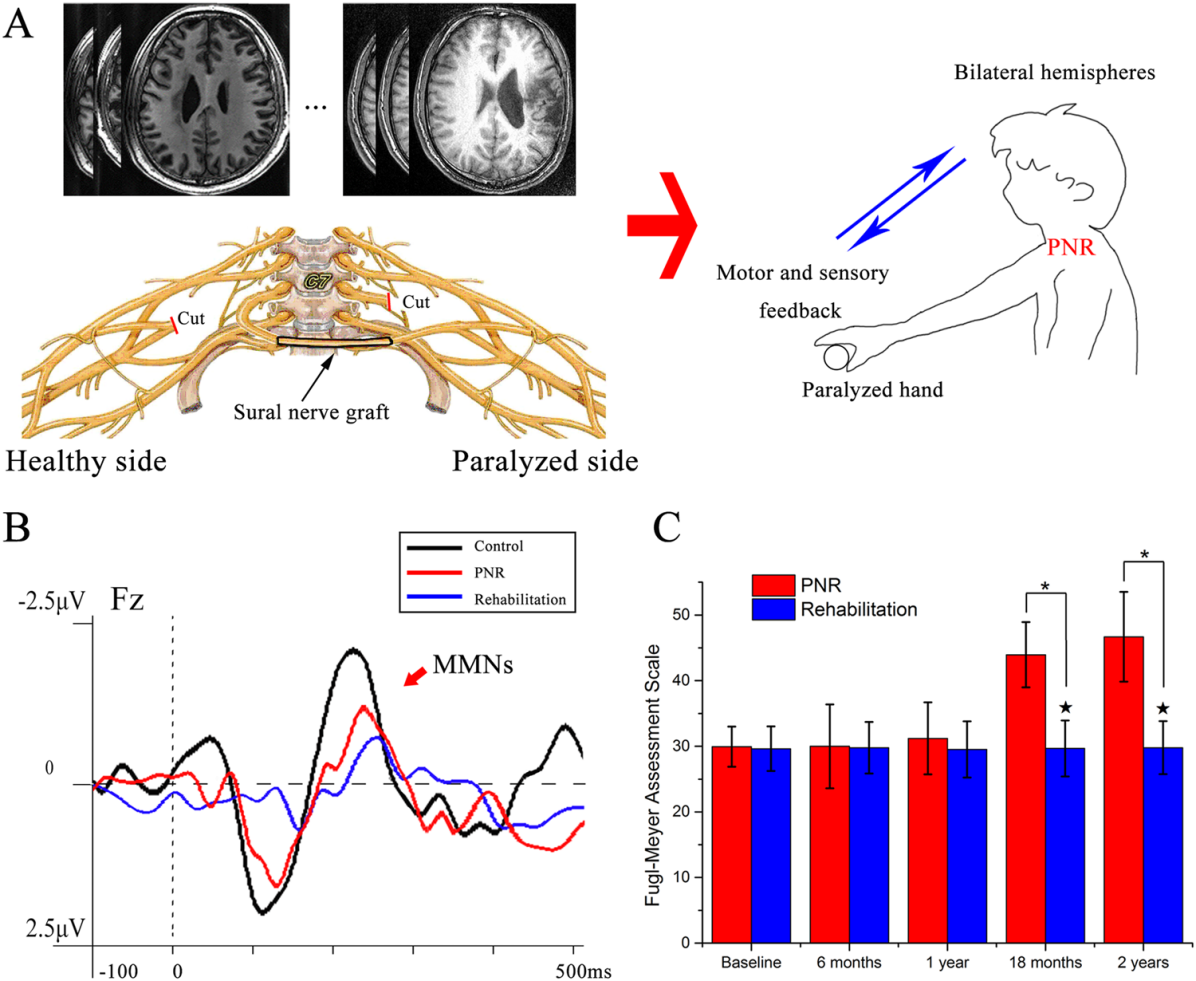
(**A**). After modification, the motor fibers from the contralateral C7 nerve (health side) allows external information input to the paralysed hand. This particular artificial neuropathway could induce interhemispheric plasticity from motor-sensory feedback during the recovery period after the peripheral nerve rewiring (PNR) procedure. (**B**) The preattentive information processing reflected by the mismatch negativity (MMN) in central neurological injury (CNI) patients after PNR was nearly normal. (**C**) Percentiles of Fugl-Meyer assessment scale scores with mean and SD. ^★^Significant differences between PNR and rehabilitation groups, P < 0.05; *Significant differences between pretreatment and posttreatment values, P < 0.05.

The Fugl-Meyer (FM) Assessment Scale scores for the two latest sessions (18 months and 2 years) showed significantly better performance in the PNR plus rehabilitation group (43.95 ± 4.98, and 46.68 ± 6.84) than the rehabilitation only group (P ≪ 0.05; group t-test). Further analyses revealed that the scores were significantly higher at 18 months (29.68 ± 4.27, 29.79 ± 4.04, P ≪ 0.05; paired t test) and 2 years (P < 0.05; paired t-test) than at pretreatment (29.95 ± 3.06, baseline measurement) and other times in the PNR plus rehabilitation group (Fig. 2C). The motor and sensory function of the unaffected hands remained normal.

The average Modified Ashworth Scale (MAS) score in the PNR plus rehabilitation group was significantly lower than that of the rehabilitation-only group. It suggested that spasticity in all joints was reduced to a lower level in the PNR plus rehabilitation group. However, further investigation of this observation is not included in the present study.

In addition to the pairwise comparisons, we calculated the Pearson correlation coefficients between the behavioural assessment results and the amplitudes of the MMN components. There was a significant correlation between the changes in the Fugl-Meyer Assessment Scale scores and in the amplitude of the MMN (r = 0.73, P < 0.05). No correlations were identified between the FM scores and the latencies of the MMN components(p > 0.1 in all cases). Additionally, there were no correlations between the MAS scores and MMNs (p > 0.1 in all cases).

### Nerve Conduction Tests of the Regenerated Contralateral C7

At the 18 months to 2-year follow up after surgery, compound muscle action potentials (CMAP) from forearm extensor on the affected side could be elicited by stimulating the graft nerve at the neck in all 19 subjects of PNR plus rehabilitation group. The mean latency of CMAP of forearm extensor muscle was 9.83 ± 0.61 ms, and the mean amplitude was 0.67 ± 0.11 mV. The result reflected a connection of peripheral neural pathway between the unaffected and affected upper extremities.

## Discussion

The major finding of this study was that the generation of the auditory MMN is clearly abnormal in central hemiplegic patients, indicating cognitive dysfunction in all our patients irrespective of whether they received the surgical treatment. The patients who underwent PNR surgery showed a gradual gain over the rehabilitation-only patients in the peak MMN amplitude during the 2-year follow-up, suggesting improved cognitive function together with the observed improvement in behavioural assessments.

The MMN has been used in cognitive neuroscience research and clinical science research^21, 22^. This rapidly measurable response is highly stable over time^23–25^ and may reflect a predominantly automatic process, allowing investigators to interrogate the earliest stages of information processing free of conscious effort and motivational artefacts that may confound the assessment of higher cognitive operations in clinical populations such as traumatic brain injury (TBI) patients^26^. To our knowledge, the present study is the first to directly measure auditory MMN as a reflection of cognitive function in central hemiplegic patients after peripheral neural pathway transposition. Compared with healthy normal control participants, the amplitudes of MMN were significantly lower in CNI patients, both before and after PNR surgery. Consistent with the present findings, MMN decreases also occurs in brain injury and stroke patients^11, 27, 28^. For example, Ilvonen *et al*.^11^ found that patients showed attenuated MMN amplitudes for tone duration and tone frequency changes to harmonically rich tones presented to the right ear at 4 and 10 days following the onset of left-hemisphere stroke. These investigators found that the attenuation of MMN amplitude was significantly correlated with impairment of cognitive ability after CNI. Even if these patients experienced plastic changes due to training or remediation programmes, their cognitive function could hardly be restored to the normal level. A classical idea is that damage to the cerebral cortex could indirectly lead to lesions of fornix, amygdala, hippocampus, mammillary bodies, and cingulate gyres, as well as to frontal cortex nerve cell degeneration, neuronal depigmentation, and apoptosis^29^. The functions of these brain regions include strong connections with recognition, information storage, learning and integration functions.

Importantly, we observed enhanced MMN in the PNR plus rehabilitation group compared with the rehabilitation-only group. Similarly, a study by Kaipio *et al*.^28^ also showed changes in MMN amplitude in closed head injury and stroke patients. There was evidence that changes in MMN were correlated with the recovery of speech comprehension in aphasic stroke patients^11^. Furthermore, Ilvonen *et al*.^11^ determined that the recovery of cortical auditory discrimination in aphasic, left-hemisphere-stroke patients, was significantly correlated with the change in the duration of MMN amplitude and speech comprehension performance from 10 days to 3 months after stroke, suggesting that the MMN can be used as an index of the recovery of auditory discrimination. In contrast to the MMN results of the follow-up, enhanced amplitude and decreased latency were observed in the present surgical interventional group and are well documented in the literature^12, 26, 30^. Recently, evidence has emerged suggesting that external environmental stimuli play an important role in shaping our brain. Särkämö *et al*.^17, 31^ reported that merely listening to music and speech after neural damage can induce long-term plastic changes in early sensory processing, which may facilitate the recovery of higher cognitive functions. In that study, the MMN frequency increased significantly for the patients exposed to both music and audio books than in the control group during the 6-month post-stroke period. Moreover, changes in the frequency MMN amplitude correlated significantly with the behavioral improvement of verbal memory and focused attention induced by music listening. However, most of the above studies are based on external stimuli, whereas, in the present study, the enhanced MMN in the PNR plus rehabilitation group suggests that, with the recovery of motor function after surgery, cognitive ability could also be enhanced synchronously, as reflected by the enhanced MMN related to preattentive change detection.

Although the above data provides evidence for the improvement of cognitive function after PNR, the mechanism underlying this improvement is easily explained. As mentioned above, peripheral nerve rewiring artificially establishes a direct peripheral-central connection from the internal neuropathways to the contralateral motor cortex, and could effectively rescue the motor and sensory function of the affected upper extremity after CNI by harnessing the potential of the contralateral cortex^5, 6^. The regeneration of the donor nerve fiber from the contralateral C7 nerve could promote the restructuring of the neural network around the motor areas and other functional region in the brain^19^, and could also stimulate the brain to generate synapses from neurons on the healthy side of the brain for the functional reconstruction of brain cells^6^.

Following PNR, an additional neural pathway was established between the contra-lesional hemisphere and the paralyzed upper extremity (Fig. 2A). Conceptually, it seems reasonable to assume that the motor and sensory feedback to both hemispheres was dramatically promoted, facilitating reorganization of the brain. From a cognitive point of view, the reorganization generates synergy between the motor cortex and the surrounding areas, which can significantly improve the processing of information processing from external events. In this process, the contralesional hemisphere could mobilize more effective resources, allowing improvements in information recognition, coding, analysis, and integration. Most importantly, additional efficient sensory-motor projections from the paralysed hand to the ipsilesional hemisphere were reconstructed, as indicated by the enhance behavioural performance of the paralysed hand at the 2-year follow up (Fig. 2C). Moreover, the increase of motor fibers from the contralateral C7 nerve subsequently promoted behaviour and increased the external information input from the paralysed hand. This particular artificial neuropathway clearly has the potential to induce interhemispheric plasticity from motor-sensory feedback during the recovery period after PNR surgery. It might improve the automatic information processing of external input by the brain (Fig. 2A), as in the case of the enhanced MMN in the present study (Fig. 2B).

It is generally accepted that the potential mechanism of cortical reorganization after stroke is the enhancement of functional connectivity within the brain network. For instance, Schulz *et al*.^32^ investigated task-related effective connectivity between ipsilesional parietal regions and key frontal motor areas using fMRI in well-recovered stroke patients. It showed that in addition to excellent motor recovery, the coupling pattern of the parietofrontal network was near-normal. Similarly, by electroencephalographic (EEG) examination, Wu *et al*.^33^ demonstrated a consistent relationship between ipsilesional M1-premotor coherence and Fugl-Meyer score at each of four examinations spanning the 28 days of therapy. Such research has increased our understanding of the parietofrontal network of the ipsilesional hemisphere as a prominent circuit involved in plastic changes after stroke. Therefore, cortical reorganization may be the outcome of network changes and the brain network between the ipsilateral hemisphere and surrounding areas might play an important role in the motor recovery of CNI patients after PNR surgery. Further study of this issue is warranted.

Although this work is based on a small sample of subjects, our findings have garnered the attention of palliative care experts. However, the small sample size may also explain some of our surprising results, namely, the apparent lack of correlation of event-related potentials and clinical functional recovery with cognitive function.

The present findings indicate that PNR affects the efficiency of preattentive information processing synchronously with the recovery of motor function in the paralysed arm. It also supports the hypothesis that interhemispheric information processing is required to detect a novel incoming external stimulus, providing insight into the pathophysiology of motor recovery, cognition, and behavioural symptoms. Additionally, the present data demonstrates the use of auditory MMN recordings for CNI patient assessment.

## Methods

### Subjects

The experimental group subjects in this study were all clinically diagnosed with central hemiplegia and were in the chronic stage of motor function recovery (more than 8 months after the onset of CNI). The lesions were documented by magnetic resonance imaging, which was separate from this study. Excluded from the study were patients with neurological diseases other than stroke or with neuropsychiatric or neuropsychological deficits that could potentially compromise their ability to provide informed consent or ensure compliance during the experiments.

The experimental group in this study were comprised two different treatments. Nineteen central hemiplegic subjects in the PNR plus rehabilitation group received the PNR surgical procedure and post-operative rehabilitation; the other 19 patients were recruited from the rehabilitation department at the same hospital based on the above inclusion criteria and received conventional rehabilitation therapy only. There were no significant differences in the age, sex, disease course, or disease severity of the subjects in these two groups (Table 2). In addition, the study included a normal control group that consisted of 19 healthy age- and gender-matched hospital personnel and patients’ family members who volunteered to participate in this study. The control group did not significantly differ from the patients in nationality, gender or age. The follow-up duration was 6–24 months after the recruitment. The disease course of the subjects was 14.1 ± 8.2 years in the PNR plus rehabilitation group and 15.7 ± 8.7 in the rehabilitation group. The demographic, clinical and lesion data of each group are presented in Table 2.

**Table 2:** Group Sample Sizes at Follow-ups.

Group	Gender		Education	Disease course	Lesion side		Pathogenesis			
	Male	female	(year, M ± SD)	(year, M ± SD)	LHD	RHD	Stroke	Cerebral palsy	Trauma	Others
PNR plus rehabilitation group (n = 19)	13	6	9.8 ± 3.3	14.1 ± 8.2	7	12	2	5	7	5
Rehabilitation-only group (n = 19)	12	7	10.2 ± 2.1	15.7 ± 8.7	8	11	5	7	4	3
Control group (n = 19)	11	8	11.1 ± 1.6	—	—	—	—	—	—	—

Education: the average number of years of education; disease course: time from the onset of central hemiplegia until recruitment to this study to receive an intervention of PNR or rehabilitation; LHD: left hemisphere damage; RHD: right hemisphere damage; others: other disease of central neural injury, e.g. encephalitis, intracranial tumour.

All subjects were right-handed. Audiometric testing was used to ensure that all participants had normal hearing and could detect 40-dB tones at 1000 Hz (mean ± SD = 12.8 ± 6.1 dB). This research was approved by the Institutional Review Board (IRB) of Huashan Hospital, Fudan University, China. All methods were performed in accordance with the approval guidelines and regulations, and the participants signed informed consent in accordance with the IRB approval stipulations.

### Stimuli

Ten sinusoidal tones were generated, starting at 500 Hz and increasing in frequency by 10% with each step, resulting in a tonal succession of 500 Hz, for 500, 550, 605, 666, 732, 805, 886, 974, 1072, and 1179 Hz. The tones were 50 ms long including 5-ms rise and 5-ms fade-out times. The intensity of all stimuli (standard and deviants) was 70 dB (sound pressure level).

### Procedure

**Figure 3:**
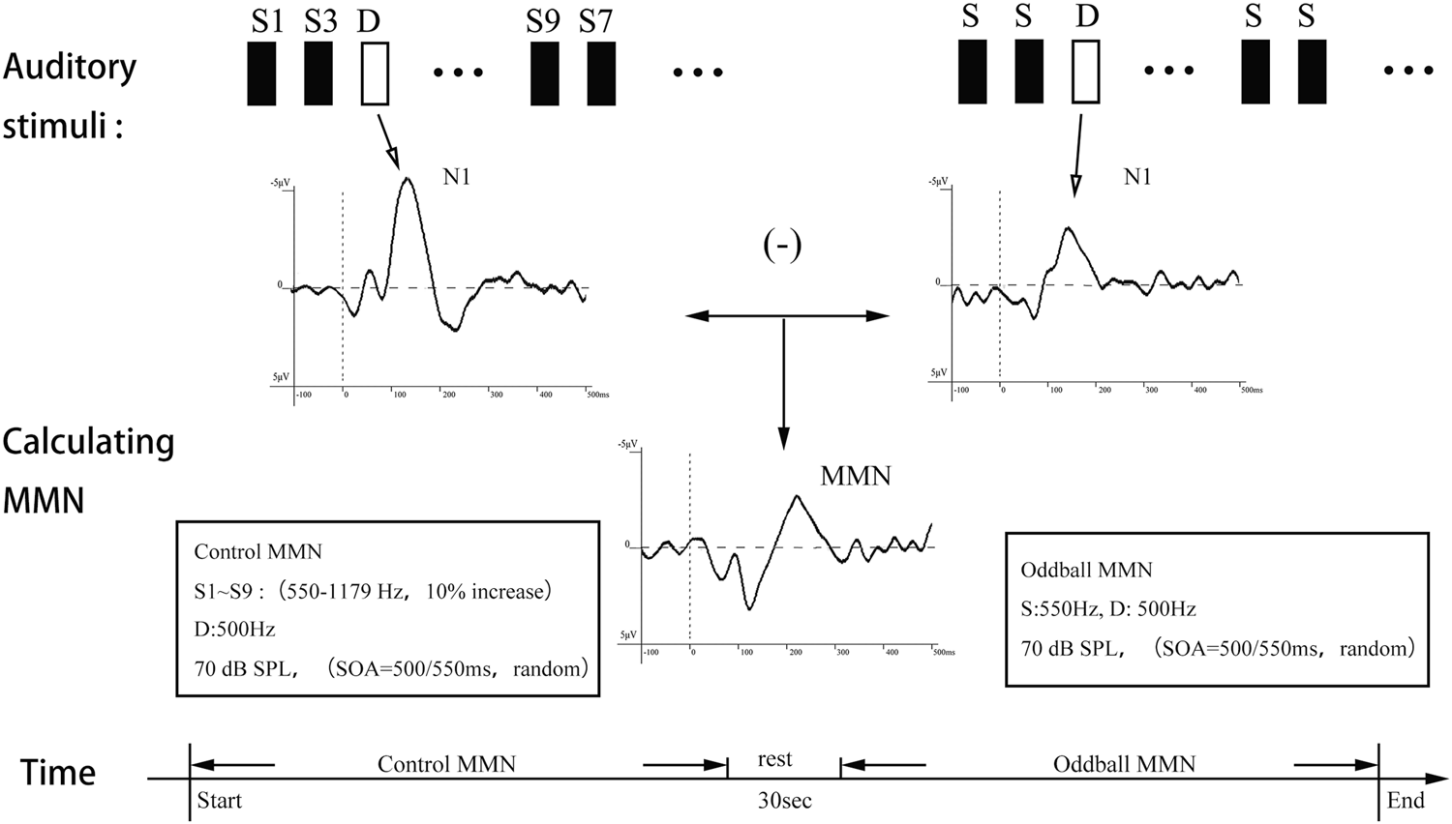
Schematic diagram of mismatch negativity (MMN) under the control condition. MMNs were calculated by subtracting the event related potentials (ERPs) of N1 in order to subtract the responses elicited by standard tones from those elicited by deviant tones in the control and oddball MMN paradigm.

The subjects were comfortably seated and instructed to watch a silent movie on a computer monitor positioned 110 cm in front of them and to ignore the auditory stimulation. Tones were sequentially presented binaurally over headphones at a sound pressure level (SPL) of 70 dB with a stimulus onset asynchrony of 500 ms. There were three blocked conditions, (a) descending deviant (500 Hz) with a 550 Hz standard, (b) ascending deviant (1179 Hz) with a 1072 Hz standard and (c) control sequence, consisting of all 10 tones. Block sequence was counterbalanced across participants. Each block consisted of 1,500 trials. In oddball blocks, deviants occurred among the standards with a relative frequency of 0.1 in a pseudorandomized fashion with the constraint that two deviants could not occur in direct succession. Deviants occurred at the endpoints of the tonal succession, the standards being their closest neighbors. In the control block, stimuli were presented equiprobably (relative frequency = 0.1) in a pseudorandomized sequence such that the set of 10 tones was multiply presented in sequence, each time in a random order, and the repetitions of tones was avoided (Fig. 3).

This procedure resulted in physically identical tones used as controls, presented in the control block, and deviants, presented in the oddball blocks, each with an equal frequency of occurrence within a block. Thus, the only difference between the two presentations was that neurons that specifically responded to the frequency of controls and deviants were less refractory for controls than for deviants, because they were presented among nine other control stimuli with a greater frequency separation in the control block, than the standard-deviant separation in the oddball block. Each experimental session lasted approximately 1 hr, including data acquisition and electrode application and removal.

### EEG recording

The EEG signal was recorded while the participants were watching a self-selected, subtitled silent film. Participants were instructed to ignore the acoustic stimuli. To control the level of attention, they were asked to answer some questions about the content of the film at the end of the experiment, including the dramatis personae, scene, and content of incident. Participants were to be excluded if they were unable to answer the question, as this indicated a lack of focus on the film. No participants were excluded for this reason.

Continuous EEG activity was collected with a Synamps 2 Amplifier for 9 electrode sites from 64 channels, F3/z/4, C3/z/4, and P3/z/4, according to the extended international 10–20 system. The reference electrode was placed on the tip of the nose. A vertical electrooculogram (EOG) was recorded from the right eye by supra- and infra-orbital electrodes, and horizontal EOG was recorded from electrodes on the outer canthi of both eyes. EEG and EOG signals were amplified from 0.1 Hz to 100 Hz at a sampling rate of 500 Hz. The electrode impedance was less than 10 kΩ throughout the experiment.

After EOG artifact correction, the EEG was segmented in epochs of 500 ms, timed from stimulus onset, and also included a 100-ms pre-stimulus baseline. Epochs contaminated with artifacts greater than ± 100 µV were rejected before the data were averaged. The EEG segments (at least 100 trials for each condition) were averaged separately for 150-ms and 50-ms stimulus conditions, and the averaged ERPs were smoothed through a low-pass digital filter at 30 Hz (24 dB/oct).

Statistical analyses were performed using the software Statistical Package for the Social Sciences, version 18.0 for Windows. The mean amplitudes of auditory MMNs were measured for the 100–250 ms time windows after stimulus onset. The amplitude differences between the groups and within the patient group between sessions were compared using repeated measures ANOVAs. Electrodes Fz, Cz, and Pz, where the MMN is largest^21^, were included in the analyses. Statistical probability from the ANOVAs was corrected using the Greenhouse–Geisser procedure. A p-value of 0.05 was considered as statistically significant.

### Behavioural Assessments

Several measures of motor recovery (reflex activity, flexor synergy, extensor synergy, movement combining synergies, movement out of synergy, normal reflexes, wrist, hand, coordination and speed) were assessed on the Fugl-Meyer (FM) Assessment Scale^34^. The Modified Ashworth Scale (MAS) was used to assess spasticity. The correlations between the changes in the MMN amplitude and in the behavioural performance was assessed using the Pearson correlation coefficient.

### Nerve Conduction Test of the Regenerated Contralateral C7 Nerve

At the 18 months to 2-year follow up after surgery, a nerve conduction test was performed to confirm the regeneration of the contralateral C7 nerve. The stimulator was placed on the graft nerve at the neck, on the side of the unaffected upper extremity. Recording surface electrodes were placed over the forearm extensor on the affected side. The width of the stimulation pulse was 0.5 to 1.0 ms and the intensity varied from 70 to 100 mA.
